# Dermatologic Effects of Selumetinib in Pediatric Patients with Neurofibromatosis Type 1: Clinical Challenges and Therapeutic Management

**DOI:** 10.3390/jcm13061792

**Published:** 2024-03-20

**Authors:** Paola Borgia, Gianluca Piccolo, Andrea Santangelo, Cristina Chelleri, Gianmaria Viglizzo, Corrado Occella, Carlo Minetti, Pasquale Striano, Maria Cristina Diana

**Affiliations:** 1 Pediatric Pulmonology and Respiratory Endoscopy Unit, IRCCS Istituto Giannina Gaslini, 16147 Genova, Italy; paolaborgia@gaslini.org; 2 Neuro-Oncology Unit, IRCCS Istituto Giannina Gaslini, 16147 Genova, Italy; 3 Department of Neurosciences, Rehabilitation, Ophthalmology, Genetics, Maternal and Child Health, University of Genoa, 16126 Genova, Italy; cristinachelleri@libero.it (C.C.); pstriano@unige.it (P.S.); 4 Pediatric Neurology, Pediatric Department, AOUP Santa Chiara Hospital, 56100 Pisa, Italy; 5 Dermatology and Angioma Center, IRCCS Istituto Giannina Gaslini, 16147 Genova, Italy; 6 Pediatric Neurology and Muscular Diseases Unit, Department of Neurosciences, Rehabilitation, Ophthalmology, Genetics, Maternal and Child Health, IRCCS Istituto Giannina Gaslini, 16147 Genova, Italy

**Keywords:** pediatric dermatology, prevention, management, selumetinib, NF1, neurofibromatosis type 1, plexiform neurofibromas

## Abstract

**Background**: Plexiform neurofibromas (pNFs) are benign neoplasms, primarily originating from Schwann cells, posing challenges in patients with type 1 neurofibromatosis (NF1) due to pain, disfigurement, compression of vital structures and potential for malignancy. Selumetinib, a MEK1/2 inhibitor, has shown promising results in treating inoperable pNFs, with clinical trials demonstrating tumor volume reduction and improved patient-reported outcomes. Despite its efficacy, dermatologic toxicities may impact the quality of life and treatment adherence. Evaluating the frequency and spectrum of such effects is crucial for effective management. **Methods**: In a four-year retrospective and prospective study, pediatric NF1 patients with symptomatic, inoperable plexiform neurofibromas (pNFs) were treated with selumetinib. Eligibility criteria included significant morbidity, pNF size exceeding 3 cm or surgical inoperability, and performance status >70%. Hematological, liver, lung and cardiac assessments established baseline health. Selumetinib, orally administered at 25 mg/m^2^ twice, was administered for two years unless a response warranting extension occurred. Cutaneous AEs were documented and graded by severity according to CTCAE v5.0, with evaluations every three to six months. The impact on symptoms and pNF size was systematically recorded, and biopsies characterized histopathological features in those patients requiring surgery. **Results**: Twenty patients were enrolled, with an average age at therapy initiation of 11.6 years. Cutaneous side effects were common, with all patients experiencing at least one and a median of two per patient. Xerosis, paronychia and acneiform rash were prevalent. Notably, pre-pubertal individuals were more susceptible to xerosis. Acneiform rash had a higher incidence in older patients and those with skin phototypes II and III. Successful management involved tailored approaches, such as clindamycin for acneiform rash and topical agents for paronychia. Hair abnormalities, including color changes and thinning, occurred, with female patients at higher risk for the latter. Paronychia presented challenges, necessitating various interventions, including surgical approaches. AEs led to treatment suspension in 20% of patients, with tumor rebound observed in 75%. **Conclusions**: According to our experience, successful management of selumetinib-induced cutaneous AEs requires tailored strategies including surgery. AEs might indirectly determine pNF regrowth due to therapy suspension. We thus emphasize the pivotal role of addressing cutaneous reactions for effective selumetinib management in pediatric patients.

## 1. Introduction

Neurofibromatosis type 1 (NF1) is an autosomal dominant neurocutaneous disorder, impacting nearly 1 in 2500 individuals globally [1,2]. It is caused by inherited or de novo variants in a gene, located on chromosome 17q11.2, that encodes for neurofibromin, a multifunctional protein involved in several cell signaling pathways, including Ras/MAPK pathway [3].

Among all the heterogeneous features related to NF1, approximately 30–50% of individuals may develop plexiform neurofibromas (pNFs) [4,5], which are benign neoplasms, primarily originating from Schwann cells yet comprising a diverse array of other cell types, including fibroblasts, perineural cells and mast cells, resulting in a heterogeneous tumor microenvironment [6]. 

Despite their benign nature, pNFs can undergo rapid growth in early childhood, particularly during the first decade of life. Such an accelerated increase can lead to disfigurement and compression of vital structures, depending on the extension and location of the tumor [7,8]. In contrast to cutaneous neurofibromas (cNFs), always benign, pNFs carry a 15% lifetime risk of transforming into malignant peripheral nerve sheath tumors (MPNSTs) [9,10,11]. While volumetric MRI has been considered the gold standard method for measuring pNFs in NF1 patients, the relatively slow growth and complex shape of plexiform neurofibromas compared to other solid tumors make it challenging to sensitively detect changes [12].

For several years, the primary therapeutic approach to plexiform neurofibromas (pNFs) has been exclusively surgical, aiming for complete or partial tumor resection. However, this procedure is frequently challenging due to significant risks of bleeding and neurologic damage, particularly in deep-seated plexiform neurofibromas involving multiple nerves. Additionally, approximately 50% of pNFs are deemed inoperable, defined as those that cannot be completely resected without risking substantial morbidity due to their proximity to vital structures, invasiveness or high vascularity.

Selumetinib, an orally administered selective inhibitor targeting kinase MEK1 and MEK2, has shown remarkably promising outcomes in individuals with different tumor types [13,14,15]. The rationale underlying its successful application in NF1 patients with pNFs is intricately tied to the loss of neurofibromin function occurring in this disease. In fact, neurofibromin downregulates Ras signaling, triggering the mitogen-activated protein kinase (MAPK) cascade. This molecular pathway subsequently promotes cell differentiation through the activation of ERK1 and ERK2, induced in turn by MEK1 and MEK2.

Trials on selumetinib involving NF1 patients have been conducted in recent years, culminating in its approval for children with inoperable plexiform neurofibroma. A phase 1 trial (AZD6244 or ARRY-142886) demonstrated tumor volume decreases from baseline ≥20% in 17 of 24 children aged 3 to 18 years, who received selumetinib twice daily at a dose of 20 to 30 mg/m^2^ on a continuous dosing schedule (in 28-day cycles) [16]. A subsequent phase 2 trial (SPRINT II) on 50 patients confirmed volumetric responses, with 66% achieving a confirmed partial response and a median reduction in neurofibroma volume of 27.9%. Additionally, 68% experienced an improvement in at least one patient-reported outcome (PRO) such as motor function, pain and quality of life [17]. These studies documented only mild to moderate adverse events (AEs), primarily involving the skin or gastrointestinal tract, and most were addressed without the need for a dose reduction [17]. Interestingly, dermatologic toxicities are often the earliest-presenting and highest-incidence AEs [18]. Although rarely life-threatening, cutaneous reactions may impact the quality of life, increase patient risk for additional infections and lead to inconsistent selumetinib administration, all of which may affect clinical outcomes [19,20,21].

We aimed to determine the frequency and spectrum of selumetinib-induced cutaneous adverse events in a pediatric cohort followed in our Pediatric Tertiary Centre, in order to assess their impact on treatment outcomes and to propose a management algorithm to address such AEs effectively.

## 2. Materials and Methods

In this retrospective and prospective observational and interventional study, individuals aged 3–18 years with symptomatic, inoperable pNFs were recruited, and selumetinib was administered on a compassionate basis. The study spanned four years from January 2020 to January 2024.

We enrolled NF1 patients (diagnosed according to the latest criteria), with pNFs causing substantial morbidity, characterized by their proximity to vital structures, major deformities, extremity lesions resulting in functional deficits, aesthetic concerns or significant pain. Additionally, pNFs needed to exceed 3 cm or be deemed inoperable through surgical means. Hematological, liver, lung and cardiac assessments established baseline health.

Selumetinib was orally administered in 28-day cycles at a dose of 25 mg/m^2^ twice a day. Treatment duration was two years in the absence of clinical response, with an additional two years if a response was observed without progression compared to baseline (defined as >20% increase in tumor volume).

Scheduled medical assessments occurred every three to six months and involved comprehensive evaluations, including history-taking, full-body skin examinations, and photographic documentation of dermatologic findings with an expert dermatologist. Weight-based dosing of selumetinib during follow-up visits was adjusted. Documented findings included recognized cutaneous reactions to selumetinib graded by severity according to the National Cancer Institute Common Terminology Criteria for Adverse Events, version 5 (CTCAE) [22]. In order to perform a more comprehensive evaluation, patients were further subdivided into pre-pubertal (<12 years old) and post-pubertal (≥12 years old) at the age of the AE. This categorization was performed based on current literature, which highlighted a higher predisposition to the development of age-dependent complications, and on our in-field experience [23].

The impact of different selumetinib regimens on symptoms or MRI-size variation of pNFs was systematically recorded. In the case of surgery for severe paronychia, biopsies were performed using traditional processing methods, including fixation in 10% formalin, embedding in paraffin and staining with hematoxylin–eosin. Additional histochemical stains, such as periodic acid–Schiff and Grocott, were employed. Immunohistochemical studies utilized CD3, CD20, MPO and CD68 PGM-1 to further characterize observed histopathological features.

Descriptive analysis included mean, standard deviation, median and interquartile range for continuous variables, as well as absolute and relative frequencies for categorical variables. The normality of distribution was assessed using the Shapiro–Wilk W test. Correlations and mean differences among population characteristics, as well as the presence and severity of AEs, were examined using chi-squared tests, Mann–Whitney U tests and one-way ANOVA. A linear regression model investigated the relationship between total treatment duration and the overall number of AEs per patient.

Furthermore, a binomial logistic regression model, with an odds cut-off of 0.5, analyzed the influence of risk factors (age at therapy initiation, gender and skin phototype, according to the Fitzpatrick scale) on the occurrence of each adverse event, providing odds ratios and 95% confidence intervals.

A significance level of *p* < 0.05 was considered statistically significant, and all analyses were conducted using the Statistical Package for the Social Sciences for Windows (SPSS version 21, Chicago, IL, USA).

## 3. Results

Twenty patients treated with selumetinib were enrolled. The average age at the start of therapy was 11.6 ± 4.63 years; sexes were equally distributed. Skin phototype (PhT) distribution was as follows: PhT I 1/20 (5%), PhT II 10/20 (50%), PhT III 8/20 (50%) and PhT IV 1/20 (5%).

The mean duration of selumetinib therapy in January 2024 was 28.9 months (range: 2.9–48.5 months). An overall AE count was conducted throughout the entire observation period.

**Figure 1:**
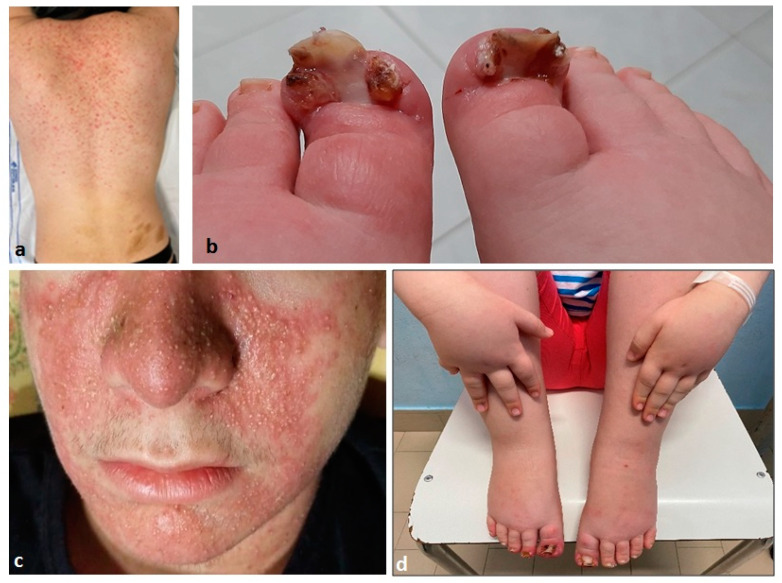
Cutaneous drug-related AE: (**a**) maculopapular skin rash; (**b**) bilateral paronychia of the first toe; (**c**) acneiform skin rash; (**d**) acral edema.

All patients (20/20) exhibited at least one cutaneous effect, with a median of two per patient (range 1–5); the results are shown in Table 1. The most frequent side effects were xerosis (14/20; 70%), paronychia (13/20; 65%) and acneiform rash (11/20; 55%). The average time from treatment initiation to cutaneous AE was 17.9 days (range 4–81 days), with the earliest manifestations being itching and hair thinning. Other AEs included hair color changes, maculopapular rash and edema of the extremities (Figure 1a–d).

Xerosis, diagnosed in 70% of patients (14/20), emerged as the predominant and swiftly occurring reaction after selumetinib administration. Patients displayed an average onset time of 0.96 ± 1.6 months. Particularly noteworthy was the heightened susceptibility of pre-pubertal individuals to this adverse event (*p*-value = 0.046), indicating a potential age-related vulnerability. Among the patients, 28.6% experienced a Grade II xerosis, while the remaining ones manifested a milder form. This symptom, not confined to specific body areas, often progressed into eczema, resembling atopic dermatitis. Patients were advised to avoid excessive bathing, prefer tepid water and constantly apply moisturizing creams, a regimen that yielded positive outcomes in all cases. Interestingly, itching accompanied this condition in 9/14 patients (64.3%, all Grade I), 3 females and 6 males, consistently responding to antihistamine oral therapy.

Additionally, 55% of the patient cohort (11/20) developed acneiform rash (Figure 1c), with an almost equal distribution between genders (6 females vs. 5 males). Such manifestations appeared at 5.05 ± 9.4 months after the onset of therapy, with seven (63.6%) classified as Grade I and four (36.3%) as Grade II. Our analysis indicated a higher frequency in older patients (*p*-value = 0.001), according to the Mann–Whitney U test. Moreover, a one-way ANOVA nonparametric test demonstrated a significant difference between the onset age and grading of acneiform rash, with a higher occurrence in older patients (*p*-value = 0.01).

Furthermore, a chi-squared age-weighted test displayed a higher incidence of this effect in individuals with skin phototypes II and III. The most commonly affected areas were the face, back and chest.

Successful treatment involved clindamycin 1% gel twice per day for at least 4 weeks for Grade I, resulting in complete resolution. Grade II patients experienced significant improvement with doxycycline 2.2 mg/kg once per day for at least 4 weeks, except for one patient who discontinued selumetinib after 16.7 months due to significant psychosocial impacts from the cutaneous reaction.

Hair abnormalities included lighter hair color/depigmentation in 3 patients (15%) and hair thinning observed in 11 patients (55%). The latter manifested across a spectrum of phenotypes, ranging from brittle hair to slow growth and reversible telogen effluvium, occurring on average after 2.72 ± 0.7 months from the start of treatment. Female patients showed a greater risk of developing such AEs, with an odds ratio of 9.5 (1.3–20.6; *p*-value = 0.04). Changes in hair color, on the other hand, occurred after a mean latency of 7.97 ± 5.75 months and were not confined to a specific region but involved the entire scalp. Notably, the Mann–Whitney U test indicated a higher incidence of this condition in younger patients (*p*-value = 0.03).

A single patient presented hands and feet edema after 4 months of treatment with selumetinib. Escalating symptoms prompted a strategic shift, including dose reduction, diuretic therapy and sodium restriction. Such interventions led to positive outcomes.

Paronychia occurred in 65% of the patient cohort (13/20) (Figure 1b). The average age at onset was 12.98 ± 4.61 years, affecting six cases (46.1%) among females and seven cases (53.9%) among males. The mean time from the initiation of treatment to the first occurrence of symptoms was 4.18 ± 2.8 months, with six patients (46.1%) experiencing Grade I forms, three patients with Grade II paronychia and four patients with Grade III. In the present study, paronychia predominantly affected the first toes, seldomly involving the others and never involving any finger.

All patients with milder forms were initially treated with topical agents such as fusidic acid and betamethasone twice per day for a period of 2–4 weeks, followed by maintenance therapy with tacrolimus 0.03% twice per day. Six out of thirteen patients (46.1%) achieved complete resolution only by topical treatment, while the others required additional interventions.

Patients experiencing Grade II and III paronychia received oral antibiotics such as doxycycline once per day for at least 4 weeks, or azithromycin 10 mg/kg three times per week (for children under 8 years of age), taking advantage of their anti-inflammatory properties. Such an approach led to complete resolution in three out of six patients. However, one patient, unwilling to undergo antibiotic therapy, opted for nail avulsion. The remaining cases underwent bilateral partial matricectomy with electrocautery, a procedure that demonstrated effectiveness in all treated patients (3/3). In a case, a biopsy of the inflamed periungual site was also performed, which exhibited a significant chronic infiltrate of mixed inflammatory cells, prominently thin-walled vessels and diffuse edema upon histopathological analysis. A cultural examination was performed in case of suspected local infection, which revealed mixed bacterial flora, and infection by Pseudomonas aeruginosa in one case.

During an average follow-up of 12 ± 6.3 months after the first cutaneous AE, six patients (46.1%) experienced a relapse. Three of them presented Grade I paronychia, whereas three showed Grade III. Topical treatment was successful in four cases, one required systemic therapy and subsequent nail avulsion, and the other one discontinued selumetinib with prompt clinical benefit.

Throughout the observation period, 3 out of 20 patients (15%) discontinued the treatment due to cutaneous AEs. Specifically, paronychia was reported in two cases, and acneiform rash in another. Notably, the latter was the only individual who did not resume the medication. Another discontinuation was due to elevated serum creatine kinase levels. Interestingly 75% (three out of four patients) displayed pNF regrowth, evidenced by MRI evaluations conducted after 6 months of the altered treatment regimen.

## 4. Discussion

Neurofibromatosis type 1 is a relatively rare genetic disorder, although advances in genetic techniques have increased its prevalence in diagnosis. One of its main complications, namely pNFs, could cause disfigurement, pain, psychological distress and an altered quality of life. While surgical interventions often fall short in radically eradicating tumors, selumetinib has demonstrated efficacy in reducing the size of growing and inoperable pNFs in children with NF1. However, these therapeutic benefits come with potential dermatologic side effects, necessitating a comprehensive understanding of these effects.

Our study revealed that cutaneous adverse events are not only common but often multiple, significantly impacting clinical management. Similarly to current literature, every child in the study exhibited at least one cutaneous AE, with some experiencing up to five different ones. The primary AEs observed were cutaneous xerosis (70%), paronychia (65%), acneiform rash (55%) and hair abnormalities (70%). The frequency of these cutaneous AEs was overall in line with that observed in phase 2 of the SPRINT trial [24], although we detected a higher prevalence of paronychia (65% vs. 50%).

Consistent with previous findings, we found prominent age-related differences in cutaneous AEs [23,25], with younger children being more prone to experience cutaneous xerosis and hair depigmentation. Post-pubertal patients over 12 years old were more likely to present with acneiform rash, often of a higher grade. This age-related variance may be attributed to changes in skin characteristics, such as sebaceous gland maturation or alterations in skin flora, which occur during puberty.

Consistent with adult studies, we also observed that individuals experiencing acneiform rash predominantly belonged to skin phototypes II and III. This difference may be imputable to the increased vulnerability of lighter-skinned individuals to the harmful effects of ultraviolet radiation (UVR). The impact of MEK inhibition on keratinocytes’ homeostasis could potentially aggravate the effect of UVR, leading individuals with lighter skin phototypes to manifest acneiform rash more frequently [26].

Hair abnormalities, including lightening of hair color and hair thinning, were observed in a significant proportion of patients (70%). The mechanism of such events can be explained by the role of the MAPK pathway in promoting hair pigment production and in protecting the hair follicle from immune-mediated damage during the catagen stage, as observed in experiments conducted on EGFR-null mice [27]. Furthermore, our study discovered a significant association between female gender and the occurrence of hair abnormalities. We postulated that the increased tendency of females to manipulate their hair, including the frequent use of tension-inducing hairstyles and sometimes trichotillomania, may play a significant role in the development of hair fragility under selumetinib [28].

Paronychia, impacting a substantial percentage of patients (68.4%), demonstrated a noteworthy recurrence rate (76.9%). This aligns with prior pediatric studies reporting paronychia incidences between 31.6% and 51.2%, surpassing rates seen in adults. The higher physical activity level in children may contribute to recurrent nail trauma, a potential causative factor. Paronychia emerged as a primary factor necessitating modifications in selumetinib treatment. Notably, patient characteristics, including gender, phototypes and age at therapy initiation, showed no significant correlation with paronychia.

The literature suggests parallels between selumetinib and EGFR inhibitors, both targeting the Raf/MEK/Erk MAPK cascade. EGFR inhibition induces premature keratinocyte differentiation, apoptosis and chemokine release, leading to inflammatory responses [27,28,29,30,31]. Our patient’s histologic findings align with EGFRIs, showing abnormal epidermis, parakeratosis, prominent dermal vessels and chronic inflammatory patterns in an edematous stroma. This supports a non-specific immune activation mechanism rather than opportunistic infection in the disease’s etiology.

However, histopathological features lack specificity, sharing similarities with other forms of chronic paronychia like juvenile ingrown toenails and those induced by mechanical or chemical factors. The diverse mechanisms underlying chronic paronychia forms result in varying degrees of inflammatory activation, contingent on clinical stages.

Our study revealed that cutaneous adverse events related to medication not only significantly impact patients’ quality of life, but also can reduce the effectiveness of therapy. Treatment adjustments, such as dosage reduction or temporary discontinuation, were performed in 20% of patients, which was lower than the 28% observed in the SPRINT study. Notably, permanent discontinuation occurred in only one patient (5.26%), compared to 10% in the SPRINT study [32]. Patients experiencing altered treatment due to skin AEs displayed a statistically significant incidence of increased pNF size on MRI compared to those adhering to the prescribed regimen, underscoring the importance of addressing and promptly treating skin toxicities. Despite this, evidence-based approaches for managing selumetinib-induced dermatologic toxicities in pediatric patients remain scarce. While treatment strategies for EGFRI-induced AEs may offer insights, their applicability to the pediatric setting is limited.

Based on a literature review, we proposed an algorithm for the timely and effective management of cutaneous AEs triggered by selumetinib in pediatric patients, to harmonize treatment adherence with quality of life. The results are summarized in Table 2.

Therapeutic management strategies for paronychia mainly consist of preventive approaches aiming to minimize skin inflammation and periungual trauma, which should be started with the initiation of selumetinib therapy. Such an approach includes antiseptic soaks with chlorhexidine for 10–15 min 3–4 times per week; wearing comfortable shoes; avoiding aggressive manicuring, onychophagia and onychotillomania; and using nail lacquer to prevent nail fragmentation. For the treatment of Grade I CTCAE paronychia in children, we recommend the use of a topical corticosteroid with medium potency along with an antibiotic as an initial treatment (e.g., fusidic acid plus betamethasone valerate cream).

Once the acute phase has resolved, we recommend transitioning to the use of tacrolimus ointment 0.03%, which provides a beneficial alternative to corticosteroids, without any long-term adverse effects, such as skin atrophy and secondary skin infections [33]. For Grade II CTACE paronychia or relapse after nail surgery, oral antibiotics represent a valid therapeutic option [34]. Specifically, we suggest doxycycline for patients over 8 years old and azithromycin for younger children. A small percentage of patients (3/13; 23%) underwent surgical treatment (partial matricectomy with electrocautery), with a positive outcome. This technique affords immediate amelioration of the ingrown toenail condition, directly addressing and alleviating associated pain and discomfort. Electrocautery ensures targeted ablation of the nail matrix, curtailing the probability of nail regrowth and thereby substantively diminishing the recurrence rates of the condition. Executed typically as an outpatient procedure under local anesthesia, partial matricectomy is a minimally invasive approach, promoting a swift and generally well-tolerated recovery that allows patients to promptly reintegrate into their daily activities [35].

Proactive measures to address acneiform rash should aim at reducing skin inflammation and be initiated concurrently with selumetinib therapy [36]. These measures encompass the regular application of adequate sunscreen on exposed body areas every two hours when outdoors, coupled with the widespread use of an emollient on the skin.

In instances where pediatric patients exhibit Grade I CTCAE acneiform rash, it is advisable to commence treatment with a topical antibiotic, specifically clindamycin 1% gel, for a minimum duration of six weeks. Clindamycin, a well-established component in acne therapeutic regimens, effectively alleviates both inflammatory and non-inflammatory lesions, showcasing broad-spectrum applicability in acne management. In cases of Grade II CTACE acneiform rash, the recommendation involves administering oral antibiotics, such as doxycycline or azithromycin, for a minimum of four weeks. Doxycycline plays a pivotal role in acne management, offering dual benefits with anti-inflammatory properties and antibacterial activity that target key pathogenic factors in acne development. Additionally, it inhibits the activity of matrix metalloproteinases (MMPs), enzymes contributing to tissue breakdown and exacerbating inflammatory responses in acne. Azithromycin is specifically prescribed for pediatric patients under the age of 8, capitalizing on properties proven effective in addressing paronychia. In the case of Grade III acneiform rash, it is recommended to discontinue the use of selumetinib until the rash improves to Grade I. Moreover, obtaining bacterial/fungal cultures is crucial if infection is suspected.

## 5. Conclusions

Our four-year retrospective study on selumetinib management in pediatric NF1 patients with inoperable pNFs reveals a critical need for comprehensive understanding and proactive handling of cutaneous reactions. The dermatologic spectrum, including xerosis, paronychia, acneiform rash and hair abnormalities, reveals the multifaceted nature of selumetinib-induced toxicities.

Moreover, our findings emphasize the significant impact of cutaneous reactions on patient quality of life and compliance with the treatment, necessitating tailored therapeutic strategies. Successful management approaches, such as clindamycin for acneiform rash and topical agents for paronychia, are crucial components for reaching effective selumetinib administration. Our study further elucidated the challenges posed by paronychia, requiring diverse interventions, including surgical measures. Importantly, AEs prompted treatment suspension in 20% of patients, with associated regrowth risk, thus underscoring the critical link between dermatologic toxicities and treatment outcomes.

In conclusion, our experience provides interesting insights into the evolving landscape of selumetinib therapy in pediatric NF1 patients, along with the need for further research in addressing and managing cutaneous reactions and targeting selumetinib-induced paronychia.

Our findings provide an effective foundation for refining treatment strategies, optimizing patient care and guiding future research aimed at enhancing the overall effectiveness of selumetinib in the management of inoperable pNFs in pediatric NF1 populations.

## Figures and Tables

**Table 1:** Patients’ dermatologic adverse events, CTCAE grading, mean onset time and relapse onset time.

Cutaneous Adverse Events	N. Patients (n, %)	Time from the Start of Selumetinib to AE Onset (Months) (m ± SD)	CTCAE Grading
Cutaneous xerosis	14 (70%)	0.96 ± 1.6	Grade I: 14 (100%)
Paronychia	13 (65%), 10 (76.9%) of whom showed relapse	First occurrence: 4.18 ± 2.8 Relapse: 12 ± 6.3	Grade I: 4 (30.7%) Grade II: 5 (38.4%) Grade III: 4 (30.7%)
Acneiform rash	11 (55%)	5.05 ± 9.4	Grade I: 7 (63.6%) Grade II: 4 (36.3%)
Hair thinning	11 (55%)	2.72 ± 0.7	-
Hair color lightening	3 (15%)	7.97 ± 5.6	-
Acral edema	1 (5%)	4	Grade II: 1

**Table 2:** Recommended treatment regimens for cutaneous AEs during selumetinib treatment. BSA: body surface area; ADLs: activities of daily living; BID: bis in die; QD: quaque die (once per day).

Adverse Event	Grade 1	Grade 2	Grade 3	Grade 4
***Paronychia***	→**Local intervention**: topical fusidic acid and betamethasone valerate cream BID for 2–4 weeks, then topical tacrolimus ointment 0.03% BID for at least 6 weeks. Wearing comfortable shoes, avoiding aggressive manicuring, and diverting children from onychophagia or onychotillomania.	→**Systemic intervention**: doxycycline 2.2 mg/kg QD at least for 4 weeks **or** azithromycin 10 mg/kg QD three times per week (for children < 8 years old)	→**Surgical intervention**: Partial matricectomy with electrocautery. In case of relapse, systemic antibiotic (doxycycline or azithromycin). Consider selumetinib withdrawal until Grade 0/1	-
***Xerosis***	*Covering < 10% BSA and no associated erythema or pruritus* →Maintain skin hydration by using moisturizing creams, and if necessary, apply topical corticosteroids for a limited duration	*Covering 10–30% BSA and associated with erythema or pruritus, limiting instrumental ADLs* →In cases of more severe involvement, utilize systemic corticosteroid therapy for a limited period. Employ oral antihistamine to alleviate itching	*Covering > 30% BSA and associated with pruritus, limiting self-care ADLs* →Consider selumetinib withdrawal until Grade 0/1. Consider oral antibiotics if superinfection. Consider gabapentin treatment	-
***Acneiform Rash***	*Papules and/or pustules covering < 10% BSA, which may or may not be associated with symptoms of pruritus or tenderness* →Sunscreen SPF > 50 applied to exposed areas of body and every 2 h when outside. Application of an emollient diffusely on the skin. Consider **Local intervention**: clindamycin 1% gel BID lasting a minimum of 6 weeks	*Papules and/or pustules covering 10–30% BSA, ± pruritus or tenderness, psychosocial impact or limited instrumental ADLs, or papules/pustules covering > 30% BSA ± mild symptoms* → doxycycline 2.2 mg/kg QD at least for 4 weeks or azithromycin 10 mg/kg QD three times per week (for children < 8 years old)	*Papules and/or pustules covering >30% BSA with moderate or severe symptoms, or limiting self-care ADLs, or associated with local superinfection* →Consider selumetinib withdrawal until Grade 0/1. Obtain bacterial/fungal cultures if infection is suspected. Begin or continue oral antibiotic for 6 weeks	*Life-threatening consequences; papules and/or pustules covering any % BSA, which may or may not be associated with symptoms of pruritus or tenderness and are associated with extensive superinfection* →IV antibiotics indicated. Consider IV corticosteroids. Temporarily suspend selumetinib
***Maculopapular Rash***	*Macules/papules covering < 10% BSA* ± *symptoms (e.g., pruritus, burning, tightness)* →Oral antihistamine	Macules/papules covering 10–30% BSA ± symptoms (e.g., pruritus, burning, tightness); limited instrumental ADLs; rash covering > 30% BSA with or without mild symptoms →Add a topical corticosteroid	Macules/papules covering >30% BSA with moderate or severe symptoms, limiting self-care ADLs →Consider selumetinib withdrawal until Grade 0/1. Consider adding oral antibiotics if superinfection	-

## Data Availability

The data presented in this study are available on request from the corresponding author.
